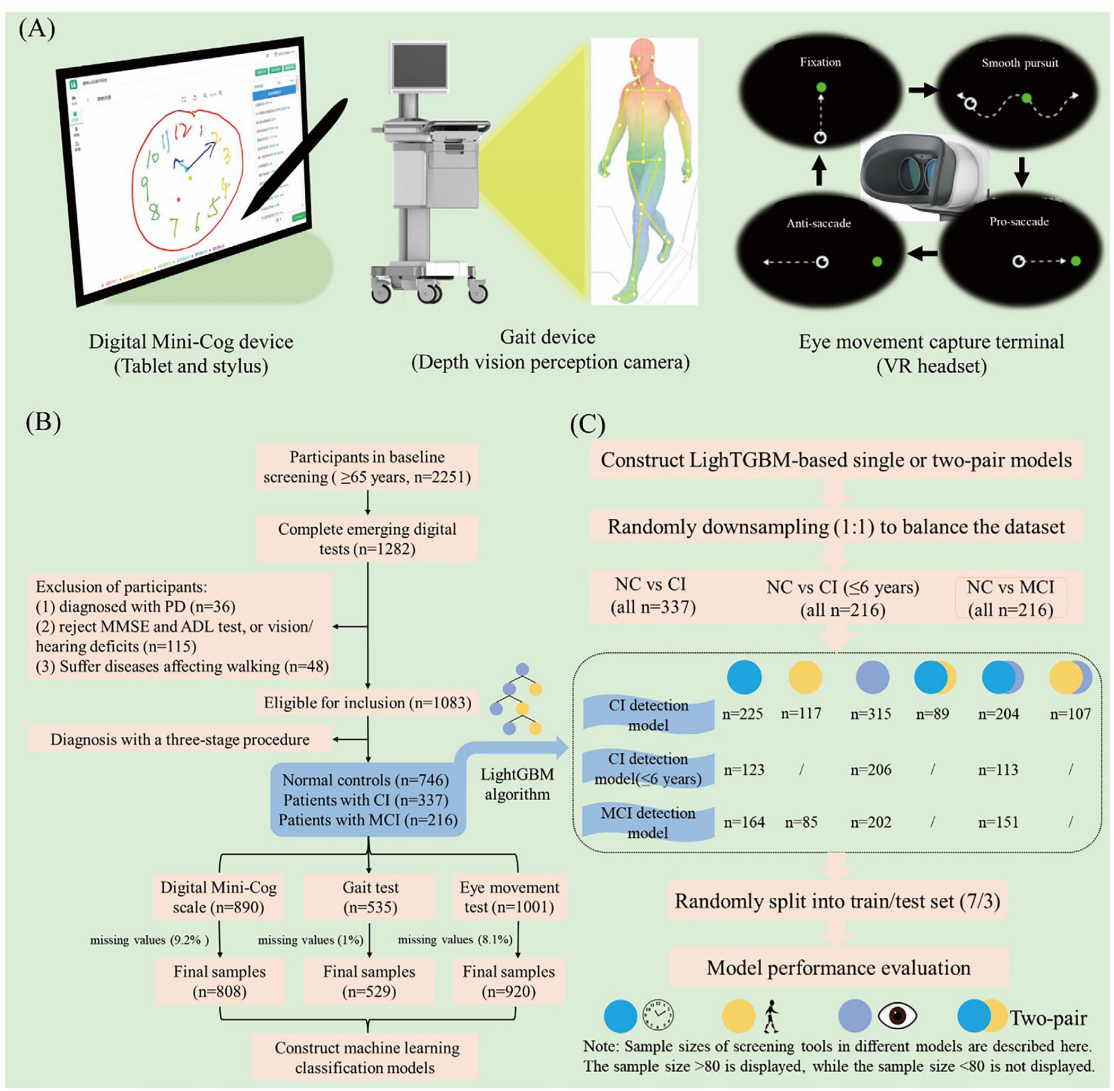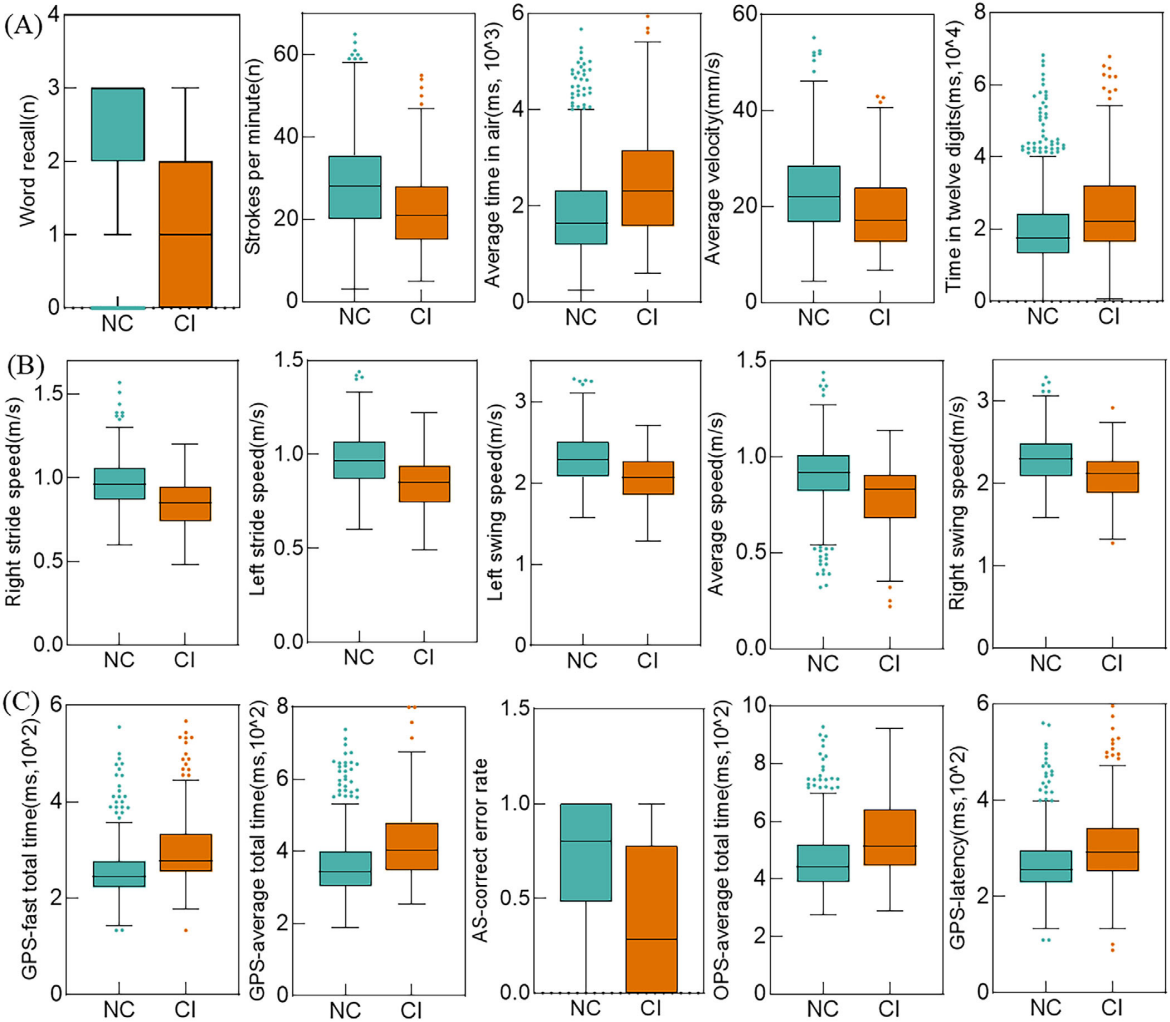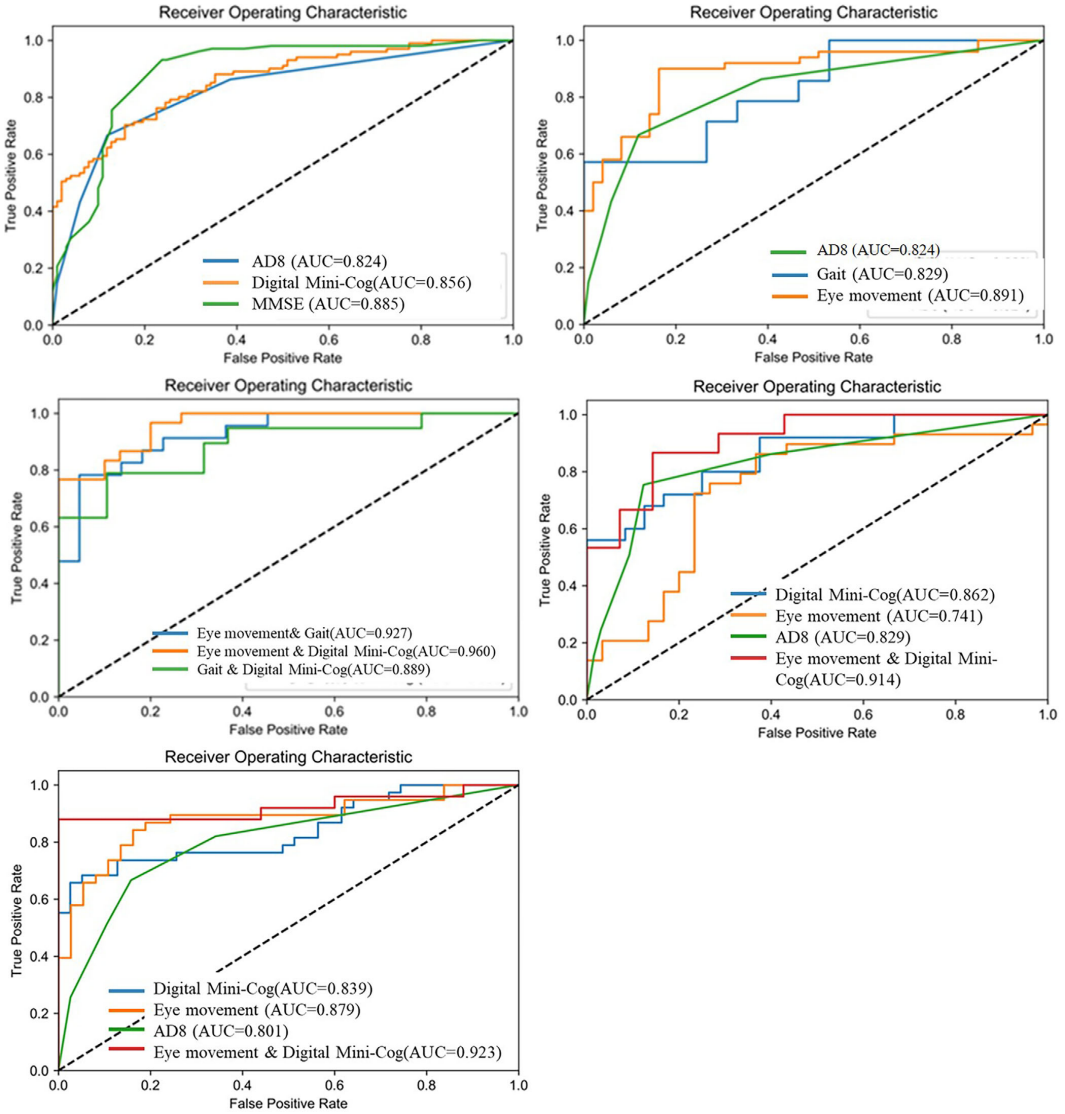# Efficacy of Emerging Digital Screening Tools in Detecting Cognitive Impairment Among Low Educational Elderly in Western China: A Community‐Based Study

**DOI:** 10.1002/alz.095764

**Published:** 2025-01-09

**Authors:** xiaonan zhang, yang li

**Affiliations:** ^1^ First Hospital of Shanxi Medical University,, taiyuan, shanxi China

## Abstract

**Background:**

This study investigated the efficacy of emerging digital screening tools for detecting cognitive impairment (CI) in a large community of elderly individuals with lower educational backgrounds.

**Method:**

This community‐based cohort study included 1083 participants (age≥65 years), 337 of whom were diagnosed with CI (64.1%≤6 years). We utilized three types of tools: traditional tests (AD8 and MMSE), a digitally improved Mini‐Cog scale, and digital behavioral tools (gait and eye movement tests). We construct LightGBM machine learning models of different digital screening tools and their combinations and evaluate their performance. The correlation between each model’s top 10 key features and cognitive domain scores was analyzed.

**Result:**

Emerging digital screening tools [AUC: eye movement (0.89)> digital Mini‐Cog (0.86)> gait (0.83)] showed promising efficacy in distinguishing CI from NC. The AUC of combination 1(digital Mini‐Cog & gait) was 0.89, and that of combination 2(eye movement & gait) was improved to 0.93. Combination 3 (eye movement & digital Mini‐Cog) achieved the highest classification efficacy (AUC = 0.96), with accuracy, sensitivity, and specificity all above 83%. In the subgroup analysis (≤6 years), the AUC for eye movement in identifying CI decreased to 0.74. Different behavioral features reflected varying impairments across cognitive domains.

**Conclusion:**

Emerging digital technologies offer objective, accurate, and efficient alternatives for CI detection, and are anticipated to replace traditional tests in community screenings. But the applicability of these tools in lower educational groups (≤6 years) should be cautious.